# Mole crab-inspired vertical self-burrowing

**DOI:** 10.3389/frobt.2022.999392

**Published:** 2022-10-10

**Authors:** Laura K. Treers, Benjamin McInroe, Robert J. Full, Hannah S. Stuart

**Affiliations:** ^1^ Embodied Dexterity Group, Department of Mechanical Engineering, University of California, Berkeley, Berkeley, CA, United States; ^2^ Biophysics Graduate Group, University of California, Berkeley, Berkeley, CA, United States; ^3^ Department of Integrative Biology, University of California, Berkeley, Berkeley, CA, United States

**Keywords:** bioinspiration, legged robots, biomimetics, terramechanics, granular media, burrowing

## Abstract

We present EMBUR—*EMerita* BUrrowing Robot—the first legged robot inspired by the Pacific mole crab, *Emerita analoga*, capable of burrowing vertically downward. We choose *Emerita analoga* as a model organism for its rapid downward burrowing behaviors, as it is four times as fast as the most rapid bivalve mollusk. Vertical burrowing in granular media is a challenging endeavor due to the tendency for the media to create upwards resistive forces on an intruder, even during purely horizontal motions. Our robot is capable of vertically burrowing its body in granular substrate primarily through excavation using two leg pairs, which are functionally analogous to groupings of leg pairs of the mole crab. We implement a novel leg mechanism with a sweeping trajectory, using compliant fabric to enable an anisotropic force response. The maximum resistive force during the power stroke is 6.4 times that of the return stroke. We compare robot body pitch and spatial trajectories with results from biomechanical studies of the mole crabs. We characterize the sensitivity of the robot to initial depth, body pitch and leg pose, and propose bounds on initial conditions which predict various burrowing failure modes. Parametric studies utilizing Granular Resistive Force Theory inform our understanding of robot behavior in response to leg phasing and orientation. Not only does this robotic platform represent the first robophysical model of vertical mole crab-inspired burrowing, it is also one of the first legged, primarily excavative small-scale burrowing agents.

## 1 Introduction

### 1.1 Current burrowing technologies

While mobile robots have become ubiquitous on land, in space and at sea, they have yet to make similar strides in subterranean settings (Aguilar et al., 2016). The difficulties of creating robots for subterranean settings are exacerbated by the challenges of operating in granular media: the substrate is dense, the environment is unstructured, and physical models for sand and soils are complex. Despite these challenges, subterranean robots have many promising application areas. Potential uses for these types of machines include agricultural site characterization (Fountas et al., 2020), geotechnical engineering (Martinez et al., 2021), construction and excavation (Melenbrink et al., 2020), and remote planetary exploration in regolith (Wei et al., 2021). Additionally, many organisms which locomote underground are not well understood due to the difficulty of observation below the substrate surface. Robophysical models tested in laboratory substrates can be tools to help biologists better understand the morphology and behavior of organisms of interest (Aguilar et al., 2016).

Recent years have seen growing interest in building robots to operate in granular media, with many potential modes of burrowing explored. As summarized by (Wei et al., 2021), various burrowing robots draw inspiration from biological organisms, including moles (Richter et al., 2002; Kubota et al., 2007; Richardson et al., 2011; Lee et al., 2019; Olaf et al., 2019), worms (Omori et al., 2013; Tang et al., 2015; Fujiwara et al., 2018; Isaka et al., 2019; Liu et al., 2019; Ortiz et al., 2019; Das et al., 2020; Alhart, 2021), plant roots (Sadeghi et al., 2014, 2017), sandfish (Maladen et al., 2011) and bivalves (Germann and Carbajal, 2013; Winter et al., 2014; Tao et al., 2020), among others. Several works aim to draw inspiration from mole crabs to create robots with legged locomotive capabilities; Russell (2011) created a robot with external flapping fins which, due to feathering on the return stroke, can locomote in horizontal planes of motion. Preliminary studies by Parihk et al. of a mole crab-inspired robotic burrower for vertical plane motion, and accompanying simulations, provide insight into burrow initiation, but the simple rigid leg design was identified as a major limiting factor to successful burrow formation (Parikh, 2020).

The scallop theorem (Purcell, 1977) states that reciprocal motion in low Reynolds number fluid will not result in net motion; in order to achieve locomotion, an agent must break symmetry by altering its geometry or trajectory. In granular media, this remains true as drag forces dominate over inertial effects. However, unlike fluids, granular media experiences changes in stress states or packing (Hosoi and Goldman, 2015) and the material properties themselves are asymmetric. The media tends to create upwards resistive forces on an intruder, even during purely horizontal motions, and purely reciprocal motions often result in net upward movement (Tao et al., 2020). As a result, making downward progress in granular media requires not only breaking the geometric symmetry defined by the scallop theorem, but also producing enough force advantage to overcome material asymmetry. Solutions include the design of legs with novel trajectories and/or compliance; several recent works have explored the design of compliant fins and skins to manipulate force response, primarily in horizontal motion planes. (Liu et al., 2019; Li et al., 2021). Other works overcome symmetry constraints to achieve downward burrowing by altering the local granular properties using fluidization, both in saturated soils (Winter et al., 2014) and with injected air (Hawkes et al., 2017; Naclerio et al., 2018).

For robots attempting to burrow, this symmetry constraint presents a unique design challenge, resulting in many systems which either locomote vertically-outward (Tao et al., 2020), horizontally (Maladen et al., 2011; Lee et al., 2019; Ortiz et al., 2019; Barenboim and Degani, 2020; Huang and Tao, 2022), or downward with external supports or downward loads (Richter et al., 2002; Sadeghi et al., 2014; Winter et al., 2014; Naclerio et al., 2018). Unsupported burrowing or “self-burrowing” is more difficult and less frequently achieved (Tao et al., 2020; Huang and Tao, 2022). We define self-burrowing as the capability of an agent to burrow its whole body downward, under its own weight with no external pushing or interaction. In Table 1, we summarize the existing vertical self-burrowing robots, drawing from review presented by Wei et al. (2021). Notably, most of the works utilize a drilling mechanism in combination with another propulsion mechanism, either through multi-segmented peristalsis or dual anchor methods.

**TABLE 1:** Summary of existing vertical self-burrowing robots We summarize efforts to create self-burrowing robots capable of vertical locomotion in granular media, drawing from work in Wei et al. (2021). Publication information, biological inspiration, and burrowing mechanisms utilized are indicated. Notably, very few prior works have been successful in utilizing excavative modes for vertical burrowing.

Vertical, self-burrowing robot	Institution	Bio-inspiration	Drilling	Hammer Mechanism	Dual Anchor	Peristalsis	Excavation
IDDS; (Myrick et al., 2004)	Honeybee Robotics	inchworm	×	—	×	—	—
Planetary Underground burrowing robot system; (Kubota et al., 2007)	JAXA	mole	×	—	—	×	—
Shovel type moving burrowing robot; (Kobayashi et al., 2011)	Tokyo I. T	mole	—	—	—	—	×
Planetary underground exploration robot; (Omori et al., 2013)	Chuo Univ	earthworm	×	—	—	×	—
HP3-Mole; (Olaf et al., 2019)	DLR	mole	—	×	—	—	—
IBR, IDS; (Tang et al., 2015; Zhang et al., 2019)	Harbin I.T.	inchworm	×	—	×	—	—
LEAVO/SEAVO II; (Fujiwara et al., 2018); (Isaka et al., 2019)	Chuo Univ	earthworm	×	—	—	×	—
EMBUR; (Treers et al., 2022); (the present work)	UC Berkeley	Pacific mole crab	—	—	—	—	×

A single work (Kobayashi et al., 2011) explores excavative, mole-inspired legged self-burrowing in the vertical direction. Several prototypes utilize rigid shoveling arms with various motion types (“rotating,” “swing,” and “slide” modes). Both horizontal and vertical locomotion, as well as steering control, are demonstrated for two of the prototypes. This work utilizes partially saturated substrates—which can ease burrowing in some circumstances—yet the substrate mechanics are not described in detail nor leg force profiles reported. We expand beyond this prior work by introducing a new compliant leg and characterizing its force anisotropy, both in experiments and novel models, and demonstrating the first instance of downward legged self-burrowing in dry media with this design. Our system is also uniquely bio-inspired, and presents the first robophysical model for vertical mole crab burrowing, to expand our physical understanding of its behavior. Legged self-burrowing, particularly in vertical planes of motion with dry media, was previously not achieved.

### 1.2 Biological inspiration

**FIGURE 1:**
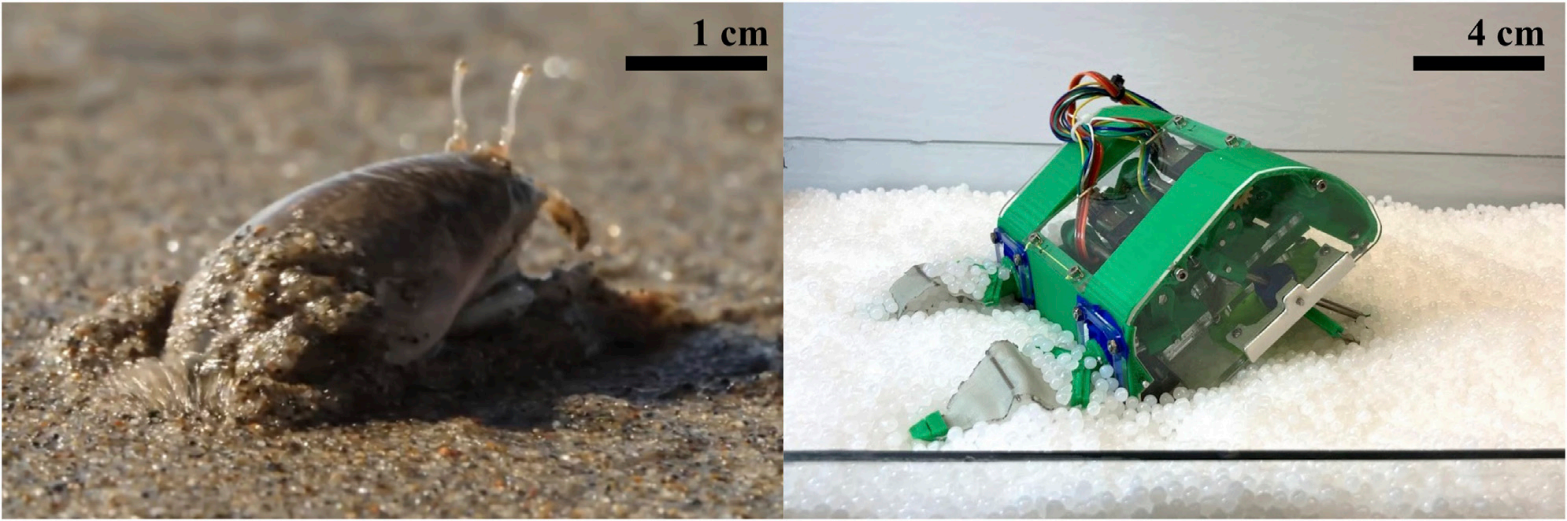
Pacific mole crab- Inspired Robot. Image of *Emerita analoga* in its natural substrate, contrasted with the mole crab-inspired robot EMBUR in the substrate used for testing. Left‐hand Image by Josh Cassidy (KQED), right-hand image by Laura Treers.

Many organisms, both terrestrial and marine, rely on burrowing in granular media for locomotion, avoiding predation or extreme temperatures, creating a place for habitation, or storing food. Mechanisms for burying and burrowing can broadly be categorized into: fracture (utilized by worms and gastropods), bulk and localized fluidization (utilized by large predators and bivalves, respectively), and excavation (utilized by crustaceans) (Dorgan, 2015). *Emerita analoga* (*E. analoga*), or the Pacific mole crab, is a decapod crustacean that resides in the swash zones along the western coasts of North America. Within these “swash zones,” or areas of shallow depth over which waves crash and recede, it is capable of burying in saturated soil in a matter of seconds, and can repeatedly unbury and rebury at different locations on the beach. At a burrowing rate of approximately 1 cm/s, the mole crabs are four times as fast as the most rapid bivalve mollusk (Dorgan, 2015; Trueman, 1970; Faulkes and Paul, 1997a; Faulkes and Paul, 1997b). This ability to rapidly and robustly burrow into challenging intertidal substrate makes *E. analoga* an ideal model system for identifying appendage design and control principles for bioinspired design. Figure 1 depicts *E. analoga* in its natural substrate, alongside the robotic counterpart introduced in this work.

**FIGURE 2:**
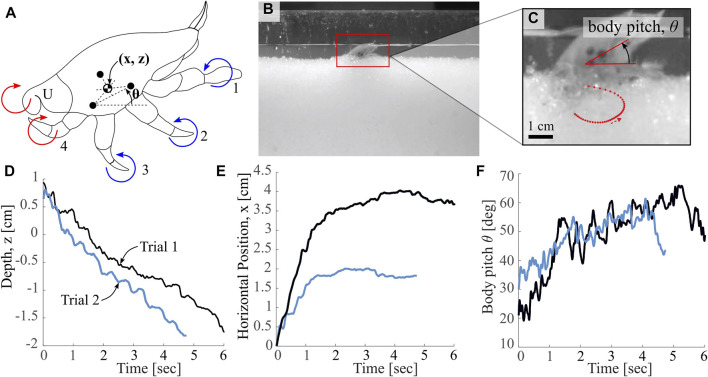
Overview of Biological Observations. **(A)** Labeled depiction of markings used for pose estimation, as well as mole crab appendages (labeled 1-4 and U, Uropods), with direction of power stroke indicated. Colors (red and blue) indicate groupings of appendages proposed in this work **(B)** Image of “ant farm” test bed used for animal experiments, as in McInroe et al. (2020) **(C)** Image of tracking points on animal, used to calculate leg dynamics, body pitch and orientation data. Red dotted curve indicates a sample leg tip trajectory from this specimen **(D,E)** Horizontal and vertical translations throughout a burrowing event for two different trials with a single crab specimen **(F)** Body pitch in the sagittal plane relative to horizontal throughout a burrowing event for the same two trials in **(D)** and **(E)**.

*E. analoga* has four leg pairs in addition to a fifth set of appendages known as uropods at the posterior of the carapace, as shown in Figure 2A. The crabs burrow along their longitudinal axis, entering the substrate with the posterior end first such that the anterior side of the body can remain above the substrate surface for filter feeding. While the crabs frequently remain burrowed close to the surface for this reason, they also frequently burrow to greater depths to avoid predation. We number the leg pairs from 1 to 4, with one referring to the most anterior leg pair and four referring to the most posterior leg pair, which is the leg pair closest to the uropods (U). Studies on Pacific mole crab burrowing have revealed details of their interleg coordination strategy, and have been conducted on mole crabs submerged in water as well as in representative media in the lab (Faulkes and Paul, 1997b; McInroe et al., 2020). The arrows in Figure 2A show how the uropods and leg pair four extend and retract in a clockwise stroke direction while leg pairs 1, 2 and 3 move in the opposite counterclockwise direction. The effects of shell streamlining have been analyzed with respect to intrusion angle, as well as the effects of varying substrate volume fraction on burrowing behavior (McInroe et al., 2018; Treers and Stuart, 2020). However, the body pitch and intrusion trajectories of the animals have not been uniquely characterized; in order to inform our robotic implementation, we seek to analyze the body kinematics of the crabs throughout typical burrowing cycles.

To quantify the characteristics of *E. analoga*’s burrowing trajectories, we measured representative translational and pitch burrowing trajectories. We used an ‘ant farm’ experimental apparatus similar to that used in Winter et al. (2012) filled with seawater-saturated granular media to facilitate imaging of the burrowing behavior with a high speed camera (Phantom v10.0) recording at 240 Hz. We used plastic granules of diameter 1.8 ± 0.12 mm as the granular substrate which allowed for limb tracking throughout burrowing. A simple penetration test, similar to that outlined by Li et al. (2013), results in a resistive coefficient for this media of *ζ* = 0.401. Black markers were painted on anatomical features of interest, including 3 marks on the carapace, and a mark at the end of each leg pair. Crabs were placed individually in the ant farm and burrowing behaviors were recorded. Details of animal experiment setup are shown in Figures 2B,C. Automated point tracking was achieved using DeepLabCut (Mathis et al., 2018).

Figure 2C includes a sample leg tip trajectory for leg pair 3 for a single leg cycle, showing the extension and retraction of the leg throughout a single stroke. We suspect that appendage retraction allows *E. analoga* to make effective vertical progress into the substrate by mitigating resistive forces during the return stroke. As shown in Figure 2D, the depth profiles for a single crab specimen for two different burrowing events are approximately linear with time, but demonstrate some oscillation throughout. In contrast, the horizontal position profiles for the same two trials (Figure 2E) increase rapidly upon burrow initiation and subsequently level off, indicating a steepening of the intrusion trajectory at some intermediate point during the burrow. The body pitch trajectories (Figure 2F) indicate an initial increase, which likely correspond to pitching up to transition from walking or scurrying to digging modes. After this initial increase, the body pitch appears to oscillate irregularly between approximately 40 and 60°.

### 1.3 Terradynamics of burrowing agents

A challenge for designing robots in granular media is the complexity of modeling substrate interactions. Several methods are utilized for predicting the resistive forces on granular intruders, ranging from computationally intensive Discrete Element Methods (DEM) to simplified empirical models like Granular Resistive Force Theory (RFT). Because DEM solves the equations of motion for every particle, it is the most reliable method but often requires on the order of days to weeks to simulate typical granular systems, rendering it impractical for large parametric design studies useful in robotic design. In this work, we choose to utilize Granular RFT to inform both design decisions as well as robot control strategies.

While RFT is a tool utilized for over 50 years (Gray and Hancock, 1955), it was introduced as a viable tool for modeling granular materials in 2009, and several works have since demonstrated its applicability to various excavation and wheeled and legged locomotion tasks (Li et al., 2013; Zhang and Goldman, 2014; Shrivastava et al., 2020). Several recent works have expanded upon our understanding of RFT, including adding velocity dependence terms (Agarwal et al., 2021), and developing models for three dimensional intrusion scenarios (Treers et al., 2021; Agarwal et al., 2022; Huang et al., 2022). Thus far, Granular RFT analyses primarily apply to dry, uniform granular media. We focus our robotic demonstrations on this type of media to simplify both the robotic implementation (i.e., not waterproofed) and modeling efforts as we parametrically study digging strategies for EMBUR in this work.

### 1.4 Overview

We present EMBUR, a Pacific mole crab-inspired self-burrowing robot which is capable of vertically burrowing its body in granular media. In Materials and Methods, we first describe the implementation of EMBUR in Section 2.1. It includes two soft elements: cuticles to keep grains out of the shell (Section 2.1.1) and flexible fabric to generate anisotropic leg forces (Section 2.1.2). A combined control method initializes and synchronizes the legs (Section 2.1.3). In Section 2.2, we introduce relevant geometric parameters and coordinate systems used to describe robot motion, as well as the methodology used for predicting leg forces with Granular RFT. Section 2.3 describes the methods used to experimentally test robot digging. In Results, we first present outcomes from RFT modeling in Section 3.1. We analyze the performance of our anisotropic legs (Section 3.1.1) and perform a parametric study to identify optimal burrowing behaviors (Section 3.1.2). We then present experimental results in Section 3.2. Results from unsupported burrowing trials demonstrate the robot’s sensitivity to initial conditions (Section 3.2.1), and we report the kinematic data for one successful robot demonstration (Section 3.2.2). In Discussion, we compare robot morphology and behavior to that of the animals (Section 4.1), discuss scaling relations between the robot operation and the animals’ natural environment (Section 4.2), and touch on potential areas for future work (Section 4.3).

## 2 Materials and methods

### 2.1 EMBUR implementation

**FIGURE 3:**
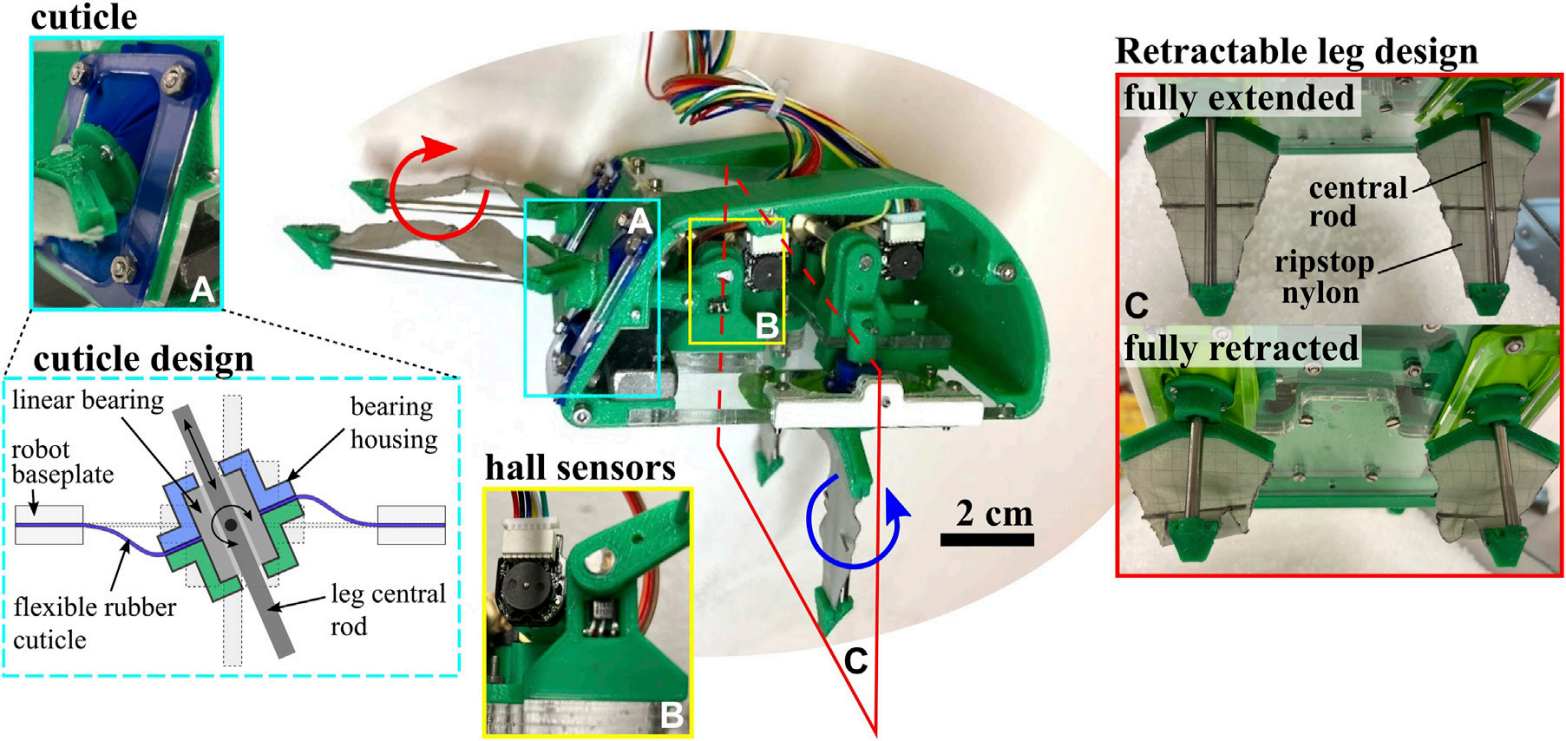
Robot Hardware Components. Image of full robot assembly, with subassembly details shown, including the cuticle design, homing hall effect sensors, and retractable fabric leg design.

Though the Pacific mole crab has five leg pairs, we functionally simplify to a robophysical model with two leg pairs in this study. In particular, these two counter-rotating leg pairs allow us to test whether downward motion can be achieved with this minimum set. A leg redundancy assumption, which allows us to simplify from five down to two leg pairs, is supported by a study in which *E. analoga* burrowed with the uropods restricted (McInroe et al., 2020). As shown in Figure 3, EMBUR consists of a shell with the approximate aspect ratio and shape of the Pacific mole crab, estimated from morphometric data of a single mole crab specimen. However, it is approximately 5 times the length of the animal; the robot shell is 10 cm long x 4.8 cm in height x 9.8 cm wide. EMBUR weighs 310 g in total. The posterior and anterior leg pairs protrude from the shell and are designed to be analogous to the uropods/fourth leg pair and the grouping of leg pairs 2/3 on the animals, respectively.

**FIGURE 4:**
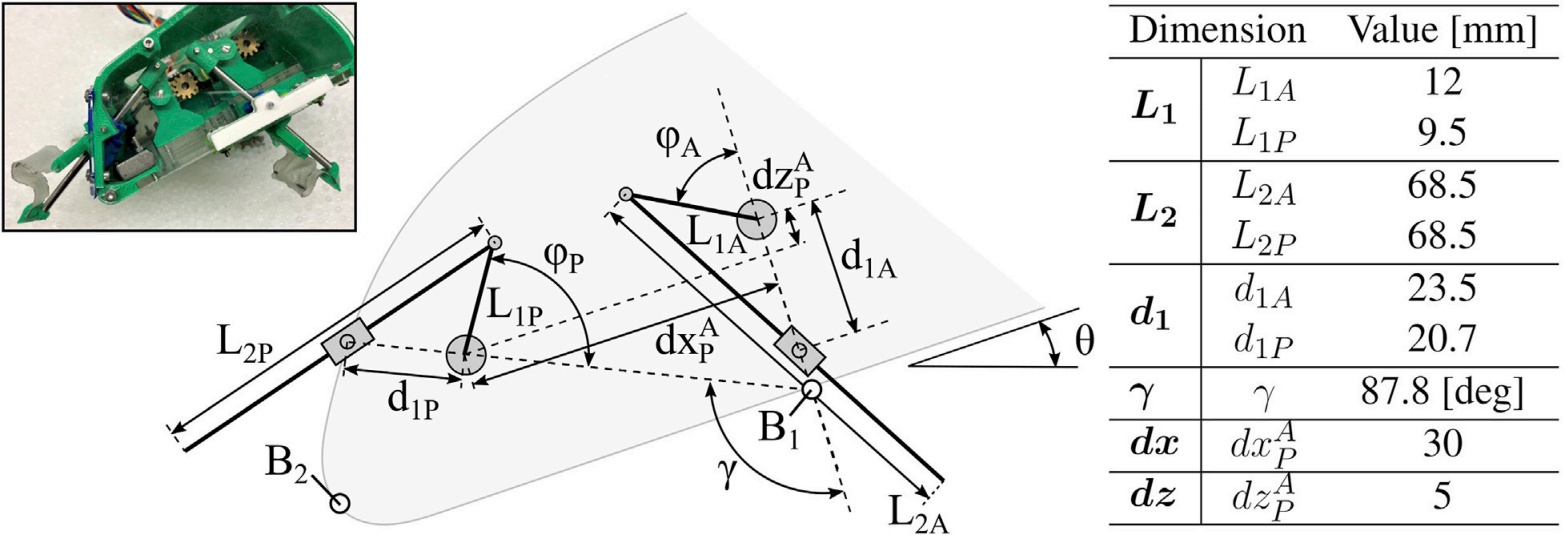
Robot Parameter Definitions. Geometric parameters of various robot components, including linkage and leg lengths, distances between pivots, and leg and body orientation angles.

Each leg pair is driven by a slider-crank linkage with a freely rotating linear bearing, the details of which are shown in Figure 4 with geometric parameters reported. The resulting trajectories of each leg pair create a cycle of insertion, a rapid sweeping or rotation of the central rod, and subsequent retraction. In this work, we refer to the rapid sweeping phase as the *power stroke*, and the remainder of the cycle stages as the *return stroke*. If each leg trajectory represents a complete cycling from 0 to 2*π*, we refer to the leg’s fully retracted state (halfway through the return stroke) as *ϕ* = 0 and most extended state (halfway through the power stroke) as *ϕ* = *π*.

A rigid central “shell” provides a housing for actuators and linkages, and is constructed of 3D printed PLA and laser cut cast acrylic. Each leg pair linkage is driven by a single brushed DC gearmotor (1000:1 micromotors with 12 CPR encoders, Pololu #1595). The transmission from the motor shaft to the shaft driving the leg linkage provides an additional 16:12 speed reduction. As pictured in Figure 3B, the shaft mounting hardware on the baseplate includes a binary hall sensor (#A3144) that triggers when the crank sweeps in front of it to provide absolute positioning. A wire tether penetrates the anterior end of the shell, and connects the motors, encoders and hall sensors to off-board motor-drive electronics and microcontroller (Arduino Due). An external power supply provides 7.4 V to the robot through the control board. Future iterations may incorporate the electronics on-board the robot to allow for remote operation. The following subsections describe the two defining compliant elements in the legs—the rubber cuticle and folding leg fabric—and leg control strategy.

#### 2.1.1 Soft rubber cuticles

As illustrated in Figure 3A, a linear bearing constrains the motion of each leg’s central rod. The linear bearing is free to rotate about pins perpendicular to the rod axis. To prevent substrate granules from entering the robot body at this mobile joint, flexible “cuticles” span the gap between the linear bearing housing and the baseplate of the robot body. This cuticle component of the robot was designed to emulate the function of the arthrodial membrane, a soft flexible tissue that is found spanning rigid joints of appendages in many arthropods (Paul, 1981). In the robot, these cuticles are made of latex rubber sheeting and clamped between two rigid layers on both the baseplate and bearing housing. The cuticle width, or distance between bearing housing and inner edge of baseplate opening, is designed to be at least one grain width across to prevent jamming of grains within the cuticle itself. We assume that the grains are too large to enter the sliding interface between the bearing and leg.

#### 2.1.2 Folding leg fabric

As the central rod of the leg extends and retracts, it expands and folds a layer of ripstop nylon, shown in Figure 3C. In order to fully deploy the compliant element, this inextensible fabric undergoes slight tensioning upon full leg extension. A thin metal crossbar is also attached to the center of the leg to prevent undesirable folding of the fabric around the central rod during the power stroke. On the return stroke the fabric is no longer tensioned by the central rod and folds in on itself. The resulting legs act as triangular “sails” during the power stroke with varying effective cross-sectional area during the return stroke. Notably, this triangular shape reduces the forces experienced at the tip of the leg as it goes deeper in the media, providing a more even distribution of force along the length of the leg during the power stroke. This type of behavior, in which cross-sectional area is intentionally altered, has been cited as a method used by both biological and robotic burrowers to break symmetry and result in net translation (Russell, 2011; Li et al., 2021; Drotman et al., 2022).

#### 2.1.3 Leg controls

Absolute positioning of the legs, *ϕ*_*A*_ (anterior) and *ϕ*_*P*_ (posterior), is performed upon startup and initialization of the robot using a homing sequence with the hall effect sensors. Prior to a digging trial, a desired phase offset between the legs is selected: $\tilde{p}={\tilde{\phi }}_{P}-{\tilde{\phi }}_{A}$ , where the ∼ overbar denotes desired, rather than actual, values. The posterior leg pair is moved to achieve this desired offset using a proportional-integral (PI) position controller. During digging, both leg pair velocities, *ω*_*A*_ and *ω*_*P*_, are separately controlled with PI controllers using feedback from the corresponding incremental encoders. The velocity set-point for both leg pairs is nominally 15.2 cycles per minute, with the two leg pairs rotating in opposite directions. EMBUR’s peak tip speed is therefore approximately 0.1 m/s, noting that *E. analoga* is estimated to produce leg tip speeds of less than 0.1 m/s (Trueman, 1970). Speeds of greater than 0.2 m/s can induce inertial effects and fluidization (Agarwal et al., 2021). Therefore, EMBUR performs predominantly quasistatic excavating behaviors in dry media.

The legs are coordinated with a strategy that uses a “leading” leg pair (anterior) and “following” leg pair (posterior). The desired leg phase offset is maintained during digging trials by adjusting the “following” leg’s desired velocity to match the “leading” leg pair using a proportional controller:

$${\tilde{\omega }}_{P}=K\left(\tilde{p}-\Delta \phi \right)+{\tilde{\omega }}_{A}$$ (1)

where *K* is a position gain value tuned to the system and Δ*ϕ* is the measured phase lag: Δ*ϕ* = *ϕ*_*P*_ − *ϕ*_*A*_. This controller maintains the desired phase offset to within approximately 20° for the tested depths in this work.

### 2.2 Burrowing simulation

**FIGURE 5:**
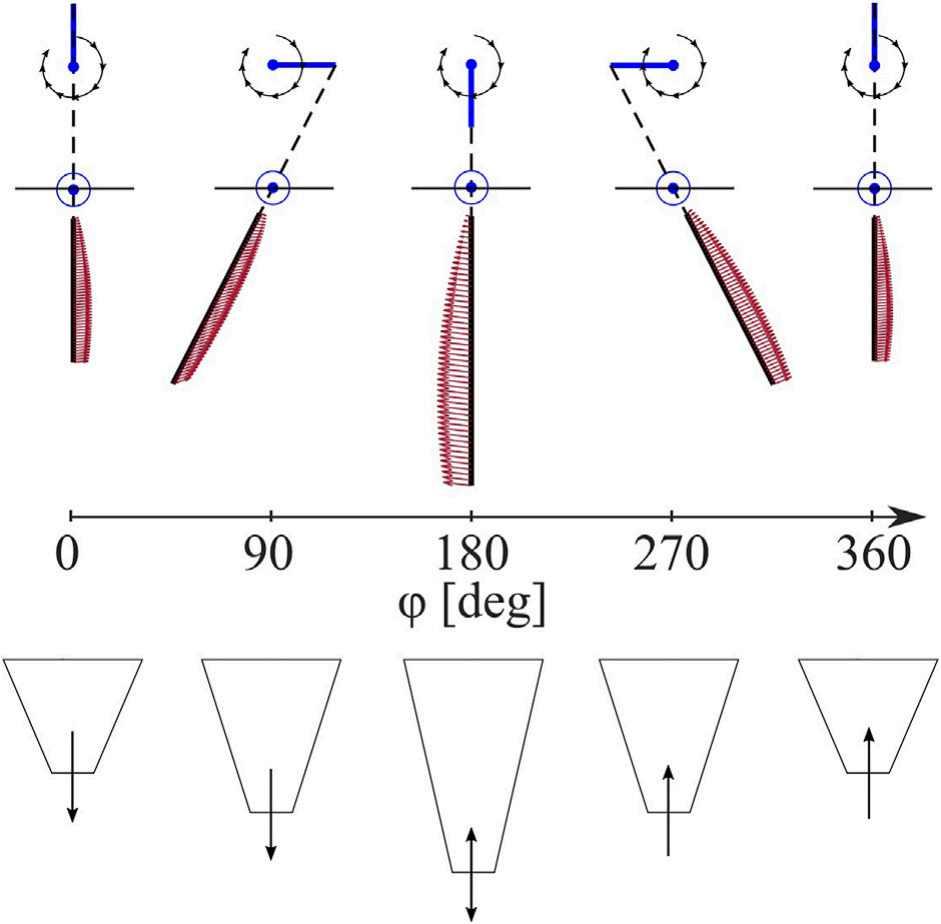
Anisotropic Leg Trajectory. Leg spatial position in black lines and force distribution in red arrows (top) and leg extension (bottom) depicted throughout a burrowing sequence from a leg pose of *ϕ* = 0–360° relative to full retraction.

EMBUR is modeled as a rigid shell fixed in space with four triangular legs (two leg pair groups) which stretch and retract in-plane throughout the legs’ sweeping trajectory. In accordance with the linkage transmission described above, we impose a fixed motion of the legs. Using 2D RFT, as presented in Li et al. (2013), allows for rapid estimation of resistive forces on both leg pairs throughout a single power stroke-return stroke cycle for various body depths and orientations. For modeling purposes, we utilize the generic 2D RFT coefficients *α*_*x*_ and *α*_*z*_ introduced by Li et al., and fit a scaling factor *ζ* to our media. We assume that leg elements that fall inside the carapace do not contribute to total resistive force. Force distribution profiles across a leg, as well as leg geometric changes throughout a single leg cycle, are depicted in Figure 5. The RFT approximations for this leg design assume that the leg is an isosceles triangle with a constant width in the frontal plane and changing length in the sagittal plane. This approximation attempts to mimic the folding and extension of the legs’ triangle-shaped fabric along the rod axis throughout each cycle.

We use this model to simulate the forces experienced by the legs as they sweep through granular media. We first simulate the anisotropic forces of a single leg pair throughout a leg stroke, *ϕ* = 0 to 2*π*. We then estimate the forces archived for a whole system, including both leg pairs. We use this method to parametrically explore the effect of placement *γ* and phase difference Δ*ϕ* of the anterior and posterior leg pairs. For both parameters, we simulate (1) the maximum moment magnitude about the COM, normalized to the mean moment magnitude throughout that cycle, and (2) the sum of work done over all elements which have a force with a component in the $-{\hat{n}}_{z}$ direction, normalized to the total work done throughout a cycle. In order to maximize force in the downward (desirable) direction, this work metric (2) should be maximized. Conversely, large moment magnitudes may destabilize robot body pitch and prevent successful burrowing, and thus we seek to minimize the moment metric (1).

**FIGURE 6:**
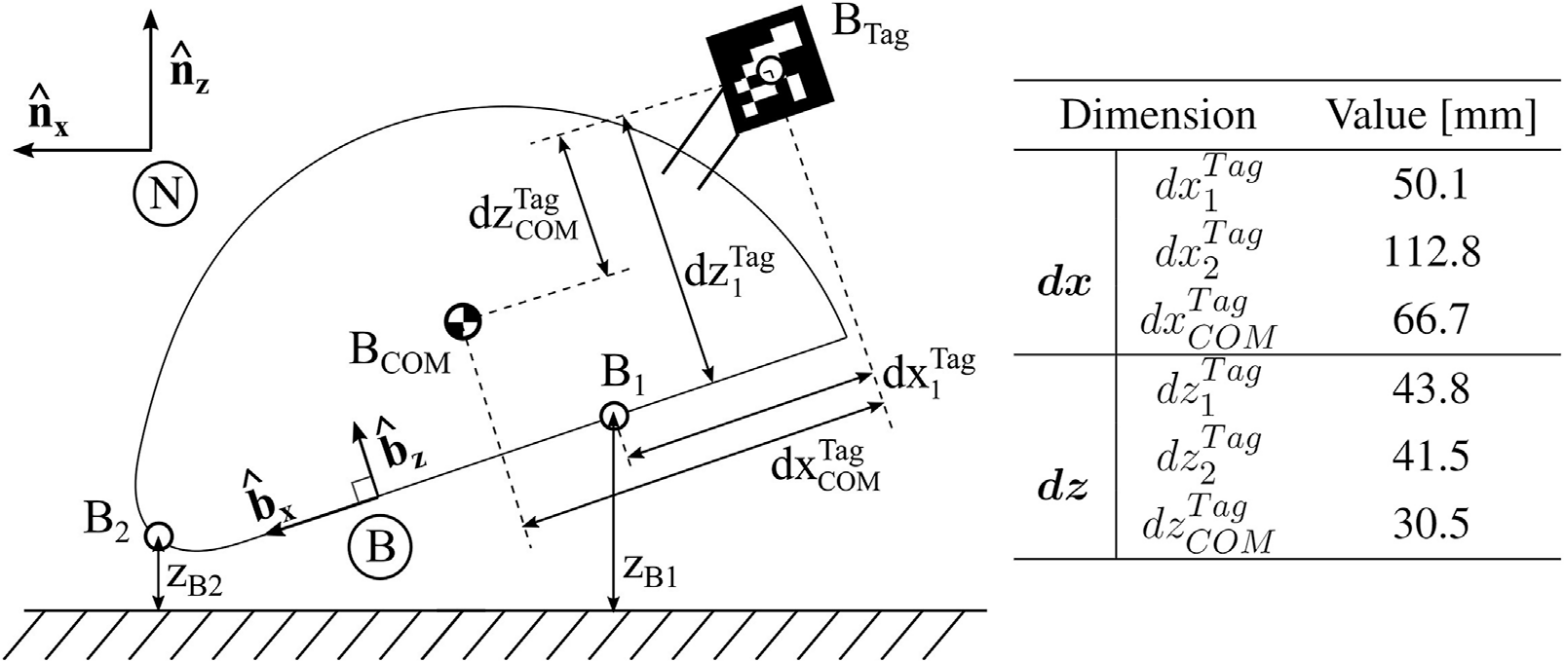
Robot Parameter Definitions. Definition of coordinate frames, including Newtonian frame N and body frame **(B)**. Relative distances between defined points on the robot body are denoted.

For describing the overall motion of the robot, we define several points and coordinates, as shown in Figure 6. The Newtonian, or fixed, frame is a right-handed orthogonal bases ${\hat{n}}_{x}$ and ${\hat{n}}_{z}$ , with ${\hat{n}}_{z}$ aligned opposite to the gravity direction. We introduce a body-fixed frame defined by the orthonormal basis ${\hat{b}}_{i}$ . The angle between the two frames, or simple rotation about ${\hat{n}}_{y}$ , is represented by the body pitch *θ*. We choose Point B1 to represent the intersection of the anterior leg with the robot baseplate in its fully retracted and extended positions. We choose Point B2 to represent the axis at the midsection of the fillet at the most posterior point on the robot shell, which is often the deepest point throughout a burrowing event. The center of mass (COM) of the robot was experimentally approximated by hanging the robot from two different carapace positions and marking the intersection of the resultant planes. This COM is located between B1 and B2 on the ${\hat{b}}_{x}$ axis, and slightly above both along the ${\hat{b}}_{z}$ axis. The locations of each of these points are listed in Figure 6, relative to an absolute position marker for motion measurements, or “Tag,” in the ${\hat{b}}_{i}$ frame. We define depth *z*_*Bi*_ as the distance of each of these points in the ${\hat{n}}_{i}$ frame from the substrate surface, with positive *z*_*Bi*_ indicating that the point is above the substrate surface.

### 2.3 Robophysical experimentation

**FIGURE 7:**
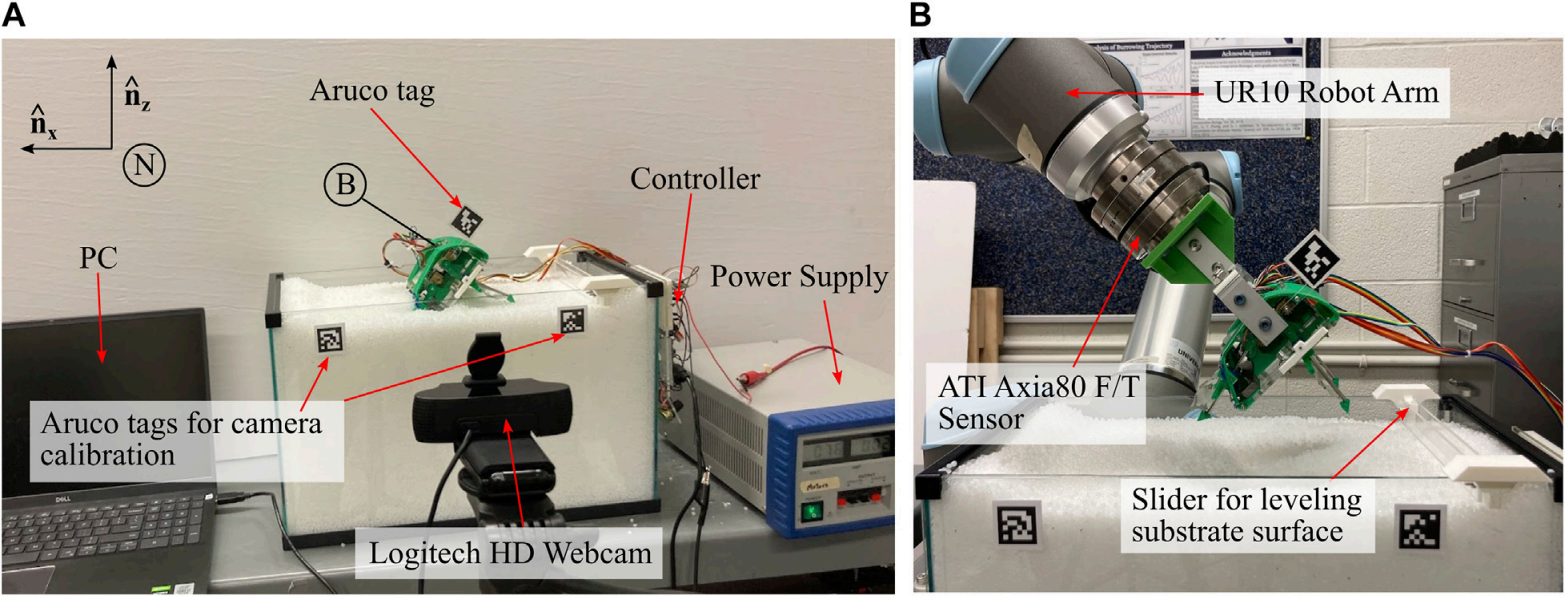
Experimental Data Collection Setup. **(A)** Setup for data collection during robot burrowing trials, with all relevant devices labeled **(B)** Setup for data collection of leg anisotropy data using the UR10 Robot Arm.

A five gallon tank (dimensions 40.7 × 21 × 25.4 cm) in the lab is filled with 3.5 ± 0.18 mm plastic pellets up to 2 cm below the top surface of tank. A penetration test, identical to that described in Section 1.2 and outlined in Li et al. (2013), results in a resistive coefficient for these plastic pellets of *ζ* = 0.111. Two sets of experiments use this setup, as shown in Figure 7.

An off-board Logitech C920 HD PRO Webcam records video frames which are then read by a python script in real time. The camera view is aligned to the substrate surface plane and an OpenCV package for Aruco tag tracking estimates robot pose. The mean vertical position of two Aruco tags mounted at the substrate surface, at either side of the tank, is subtracted from the robot Aruco position to calculate depth. The angle formed by these two tags is subtracted from the measured robot Aruco pitch. The same python script also syncs data from the Arduino Due serial port to compile encoder counts for each motor, as well as commanded phase and PWM values for post processing. Between each trial, the granular media is stirred manually and leveled to a consistent height using a slider mounted to the sides of the tank.

#### 2.3.1 Leg anisotropy

For leg anisotropy testing and for comparison to RFT modeling, the robot’s shell is mounted to a Universal Robot (UR10) arm, used to position and fix the robot in a desired location in the media. Position relative to the media is measured by the Aruco tags, and an ATI Axia 6 Axis Force/Torque sensor measures force data throughout each trial. Deviation of force data, at the onset of leg actuation after insertion, is used to indicate the beginning of a trial for syncing load cell data with the python data collection.

#### 2.3.2 Free whole body burrowing

Free burrowing is performed by first setting the initial conditions of the robot, without the legs moving, and subsequently controlling the legs and tracking the resulting body movements. A series of steps were taken before each free burrowing trial to consistently initiate the depth and orientation of the robot. After first setting the starting pose of the legs, EMBUR was placed into the media by hand. We place EMBUR in the center of the tank to minimize boundary effects ( > 5 cm from the nearest sidewalls and > 15 cm from the bottom of the tank). Using a real-time readout of depth and orientation from the Aruco tag, EMBUR was positioned to the desired pose. Initialization orientation of the body was further verified using a handheld inclinometer.

## 3 Results

### 3.1 Simulation results

#### 3.1.1 Anisotropic leg forces

**FIGURE 8:**
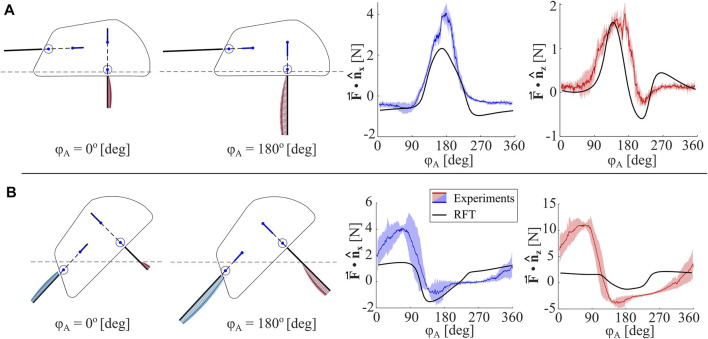
Anisotropic Leg Analysis. **(A)** Measured horizontal and vertical forces for a single leg pair (mean is denoted with a colored line and shaded region indicates standard deviation), and RFT model-predicted forces for an identical body pitch (*θ* =0°), depth (*z*_*B*1_=−0.35 *cm*), and phase (*ϕ*_*P*_ − *ϕ*_*A*_ =0). Two robot configurations, ie. two values for *ϕ*_*A*_, are shown, and location of granular media surface is indicated with a dashed line **(B)** Measured horizontal and vertical forces for two leg pairs, and RFT model-predicted forces for an identical body pitch (*θ* =45°), depth (*z*_*B*1_=1.27), and phase (*ϕ*_*P*_ − *ϕ*_*A*_ =0).

We test two different poses of EMBUR relative to the media: (1) the robot baseplate is fixed coplanar with the media surface with a depth of *z*_*B*1_ = -0.35 cm, such that only one leg pair interacts with the media, and (2) the robot is fixed at 45° with *z*_*B*1_ = 1.27 cm, such that both legs and part of the shell all interact with the media. Forces are recorded throughout several leg cycles after reaching steady-state behavior. Both the model and experimental forces in horizontal and vertical directions for (1) and (2) are compared in Figure 8.

For a single leg (1), the mean peak $\vec{F}\cdot {\hat{n}}_{x}$ during the power stroke was 4.06 ± 0.34 N, and the return stroke was −0.64 ± 0.28 N, which corresponds to an anisotropic ratio of 6.4. The robot applies more thrust in the posterior direction than the anterior direction, as intended. The RFT model predictions follow the same trends and approximate magnitudes as the experiments. For the case with both legs (2), the peak mean $\vec{F}\cdot {\hat{n}}_{z}$ during the power stroke was 11.04 ± 0.54 N, and the return stroke was −3.927 ± 0.897 N, which corresponds to an anisotropic ratio of 2.81 for the system in the opposite direction to gravity. This outcome indicates larger forces pushing the body out of the media than pulling it into the media. Note that the body is fixed, and therefore does not replicate free burrowing. There is also a notable difference between the experimental data and RFT predictions. It is suspected that phenomena not captured by RFT play a role in whole body burrowing, such as localized substrate rearrangement and static forces imparted on the shell.

#### 3.1.2 Simulated results of leg orientation and phasing

**FIGURE 9:**
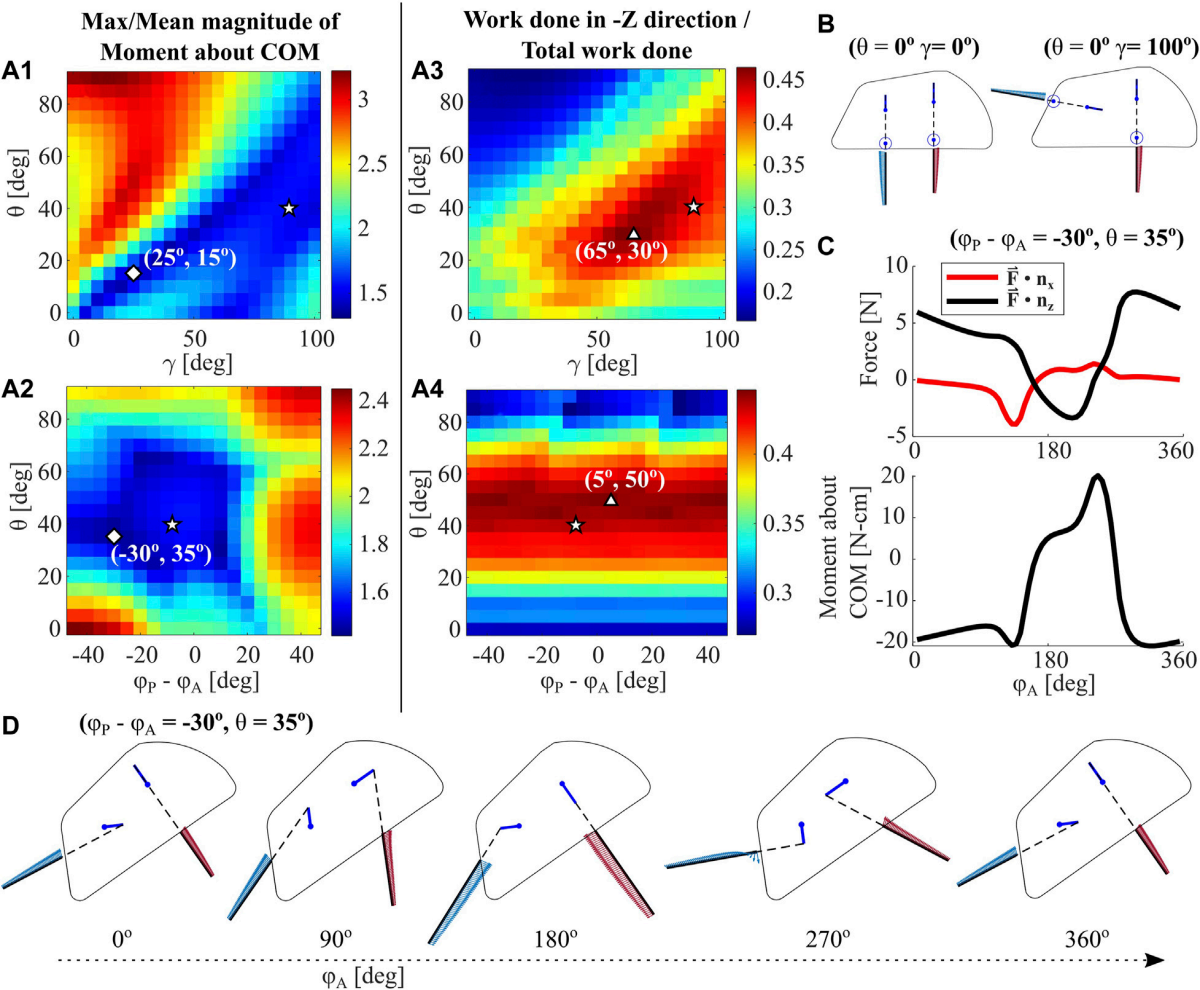
RFT Parametric Analysis. The RFT model for a single leg is used to analyze a 4-leg robot with varying body pitch *θ*, leg phase offset *ϕ*_*P*_ − *ϕ*_*A*_, and offset in leg pose *γ* **(A1-2)** Maximum moment magnitude over entire leg cycle, normalized to mean moment magnitude for each trial, plotted over body pitch, phase, and *γ*. Diamonds represent model global minima, triangles represent model global maxima, and pentagrams represent the deepest recorded burrow in experiments **(A3-4)** Work done with components in the gravity (-Z) direction, normalized to total work done over a leg cycle, plotted over body pitch, phase, and *γ*. Experimental initial conditions for the deepest measured burrow, as well as global maxima and minima, are indicated **(B)** Example leg configurations for two values of *γ* at *θ* = 0° and *ϕ* = 0° **(C)** The RFT-predicted horizontal and vertical forces over a leg cycle, for the global minimum point indicated in **(A2) (D)** Depictions of robot shell, leg pose, and force distribution throughout a single leg cycle for the minimum point indicated in **(A2)**.

In order to inform robotic design decisions around leg orientation relative to the carapace, we systematically vary the geometric parameter *γ* from 0 to 100° in 2D RFT, as shown in Figure 9A1,A3. The extremes of leg geometry tested are visualized in Figure 9B for reference. Depth of the COM is held constant at −5 cm, and phasing between leg pairs is held constant at 0°.

We then vary the input parameters phase (Δ*ϕ* = *ϕ*_*P*_ − *ϕ*_*A*_) and body pitch *θ* with a fixed leg orientation *γ* = 87.8^*o*^. The resulting moment and work metrics are plotted in Figures 9A2, A4, respectively. In each figure, we denote the global minima and maxima as predicted by RFT, in addition to the experimental values for *θ* and *γ* in the deepest recorded experimental trial, Trial ⋆ (see Section 3.2.2). While the parameters of this successful experimental trial do not align with global minima and maxima, they do fall in areas that RFT predicts to be similarly desirable for robot operation. In particular, the selected robot leg orientation implementation at *γ* = 87.8^*o*^ appears less sensitive to variations in *θ* than the optimal.

Figure 9C plots a sample RFT force and moment profile for the global minima in A2 (*ϕ*_*P*_ − *ϕ*_*A*_ = −30^*o*^, *θ* = 35^*o*^), and D shows the motion and leg force distributions for this case. Notably, *Z*-direction force remains largely positive and the work metric values in both A3 and A4 rarely exceed 0.5. However, as experimentally demonstrated in Section 3.2.2, we achieve downward motion in a vast majority of trials. The RFT model only captures leg resistive force profiles for a stationary robot body. Future work will seek to investigate additional phenomenon to more fully simulate the forces and movements experienced by this excavative robot.

### 3.2 Robophysical results

#### 3.2.1 Sensitivity to initial conditions

To systematically evaluate the effect of various initial conditions on robot burrowing, we vary both the initial depth and initial pitch of the robot while maintaining a constant velocity and leg phase. Based on preliminary experimentation, when the anterior leg pair slightly leads the posterior the body pitch remains more stable than at Δ*ϕ* = 0, so we choose a constant phase lag *ϕ*_*P*_ − *ϕ*_*A*_ = −7.5^*o*^ for all experiments. We choose four different goal initial depths: *z*_*B*1_ = -1, 0, 1, and 2 cm.

**FIGURE 10:**
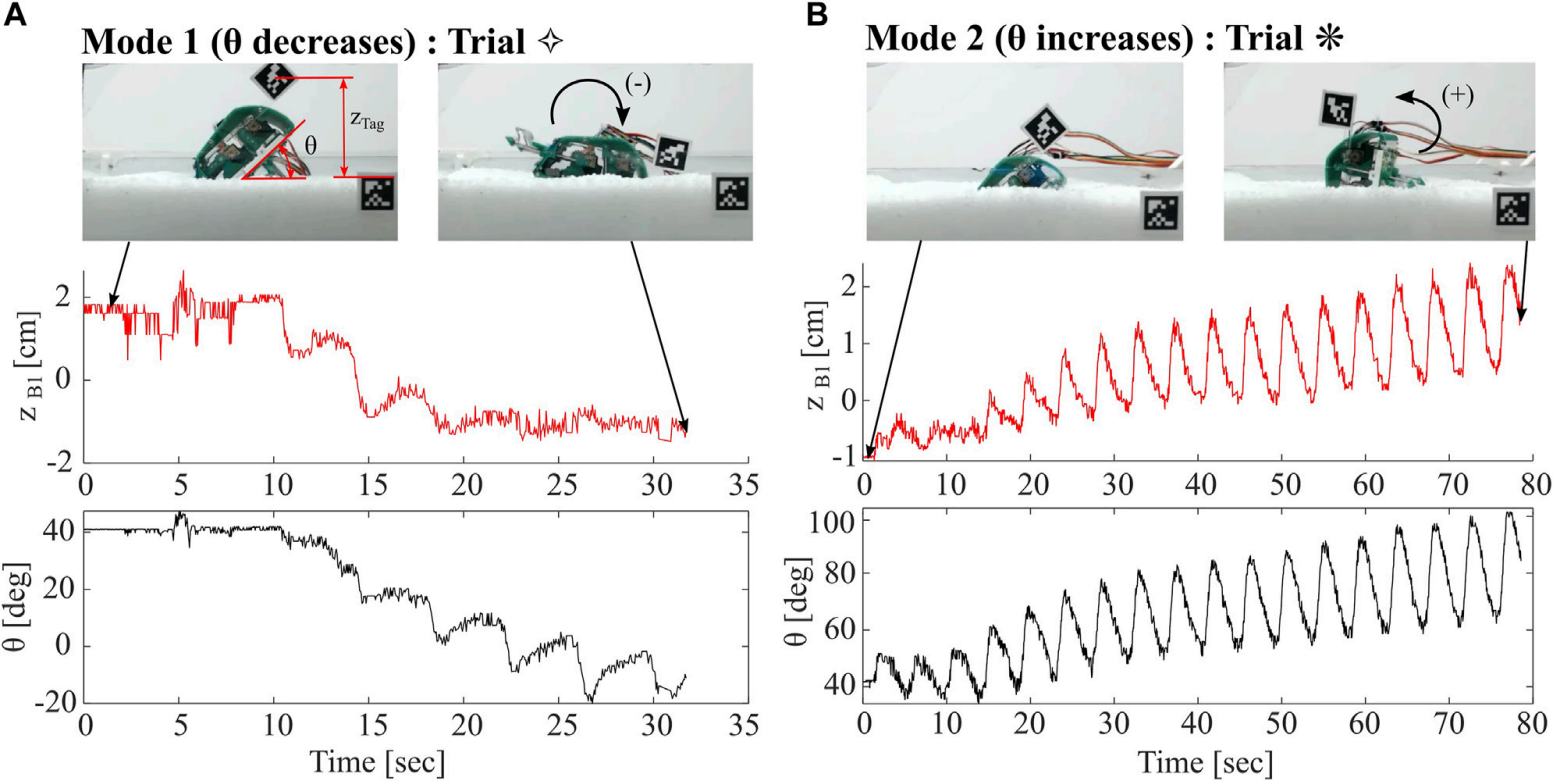
Comparison of Robot Burrowing Modes. **(A)** Mode 1, in which robot body pitch decreases from its initial condition and depth *z*_*B*1_ decreases **(B)** Mode 2, in which robot body pitch oscillates significantly, with an increase from its initial condition. Depth *z*_*B*1_ oscillates with net increase over the trial.

Tracking of body pitch throughout trials indicates the existence of two distinct modes of operation: one in which the body pitch decreases from initial, denoted as Mode 1, and another in which body pitch increases from initial, often in an oscillatory behavior, denoted as Mode 2. In both modes, the increase or decrease in body pitch is often non-recoverable in that the greater the deviation from initial pitch, the less likely the pitch is to return to its initial value. In all trials, we continue to run the robot until *θ* decreases below 0° (Mode 1), or *θ* begins to exceed 90° (Mode 2). In Figure 10, we show depth and pitch data for two of the trials with these different modes. Trial ⋄, a Mode 1 example, represents the shallowest recorded trial. Body pitch decreases below zero in a matter of three to four leg cycles, and consequently *z*_*B*1_ slightly decreases and settles at approximately −1 cm. In contrast, Trial * shows a sample from Mode 2, in which body pitch increases in an oscillatory fashion over many cycles. The minimum depth occurs at the initial condition, and *z*_*B*1_ subsequently increases as body pitch increases.

**FIGURE 11:**
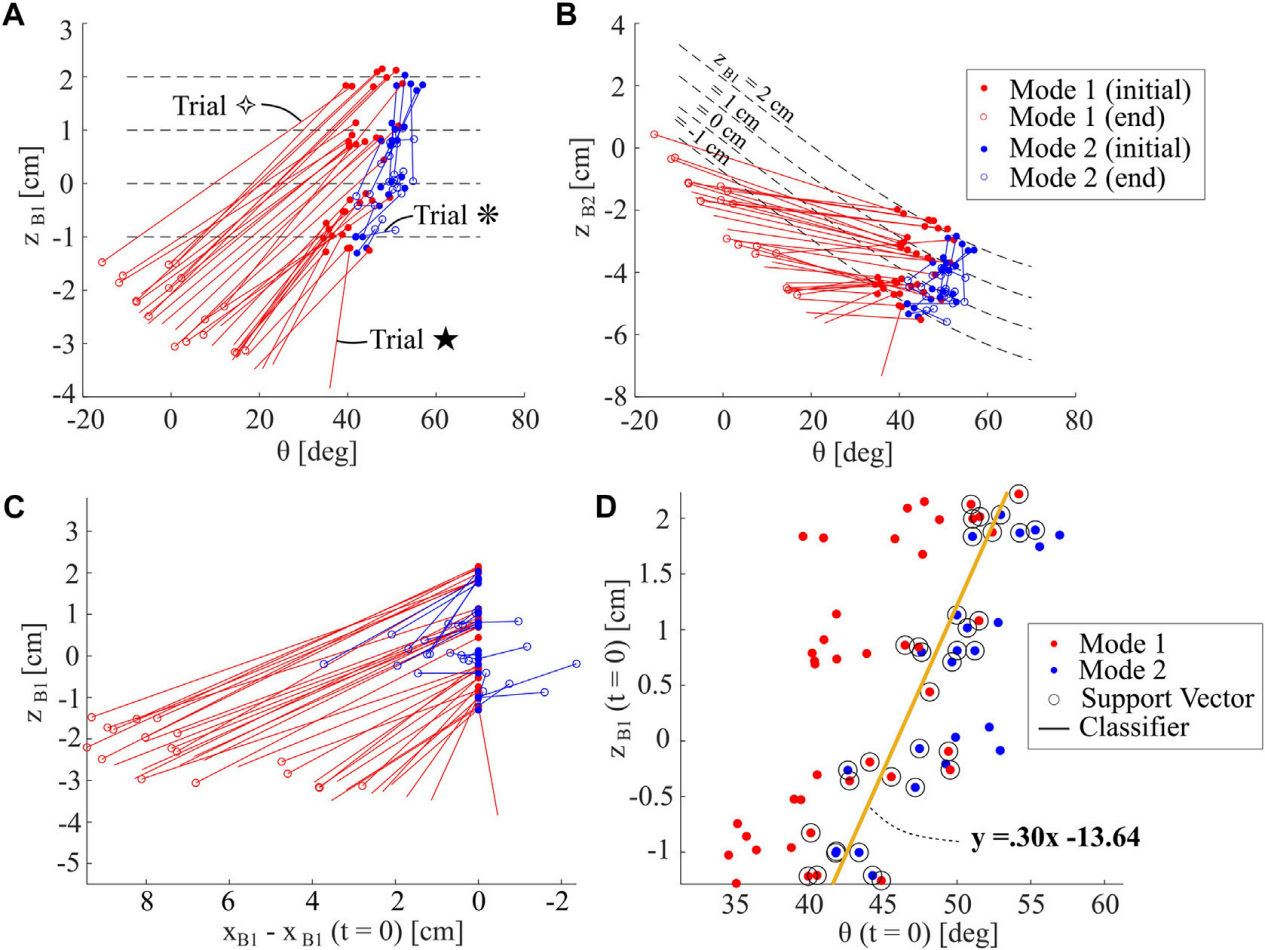
Analysis of Sensitivity to Initial Condition. **(A)** Depth *z*_*B*1_ and body pitch *θ* at both initiation and minimum depth of burrow, for many burrowing sequences. Data is separated into modes one and 2. Trials without hollow circles indicate that the Aruco tag was submerged at the minimum depth achieved **(B)** Identical data to that shown in **(A)**, but plotted over depth of Point B2 (*z*_*B*2_) on the robot shell **(C)** Depth data plotted over change in horizontal position. Note that horizontal position axis is flipped to match the direction of the ${\hat{n}}_{x}$ basis **(D)** Support Vector Machine (SVM) Analysis of initial condition data in plot **A**, with a classifier separating the two burrowing modes indicated.

Figure 11 summarizes the results from 59 trials. Red and blue lines indicate whether trials fall in Mode 1 or 2, respectively. The solid dot indicates the initial condition of a trial and the hollow dot indicates the moment of minimum depth for B1. Trials without hollow dots mean that the Aruco tag became submerged and so we are unable to measure the minimum depth. In some scenarios in Mode 2, the minimum *z*_*B*1_ value was at the initial condition. In these scenarios, we select the minimum depth after a single leg cycle has completed as the end condition, as the robot demonstrates a burrowing-out behavior. Trials indicated with symbols ⋄ and * correspond to the trials in Figure 10, and the Trial ⋆ corresponds to the deepest trial recorded; these trials are all shown in the Supplementary Video S1.

In both Figures 11A,B, the slopes of the lines connecting initial and end conditions are similar for all Mode 1 trials, while those for Mode 2 are stochastic. The trials that start with higher initial *z*_*B*1_ also have higher final *z*_*B*1_, likely due to the greater instability in body pitch when the robot is less submerged. Figures 11A,B represent the same data, but visualized for points B1 and B2, respectively. We indicate dashed lines representing equal *z*_*B*1_ initial depths, as defined by robot geometric constraints. In most trials, *z*_*B*2_ increases rather than decreases, with the exception of the most successful trials. It is the rotation of the body in Mode 1, along with the lowering of the anterior of the robot, that enables body submersion under the media. As highlighted in Figure 11C, Mode 1 burrowing also tends to result in more horizontal travel towards the posterior, except for the deepest trials.

In order to analyze the sensitivity of the robot behavior to these input conditions, we then compile the input conditions for *z*_*B*1_ and *θ*, and assign them a Mode 1 or 2. Using a support vector machine algorithm, we are able to compute a linear classifier for the data. This linear classifier, as indicated in Figure 11D, has a positive slope, indicating that the separation point between the two behavioral modes occurs at higher body pitches for greater initial depths. Because the deepest burrowing trials occur quite close to this classifier line, this analysis could ideally inform future robot control schemes, by indicating the approximate optimal regime for operation.

#### 3.2.2 Deepest robot burrowing demonstration

**FIGURE 12:**
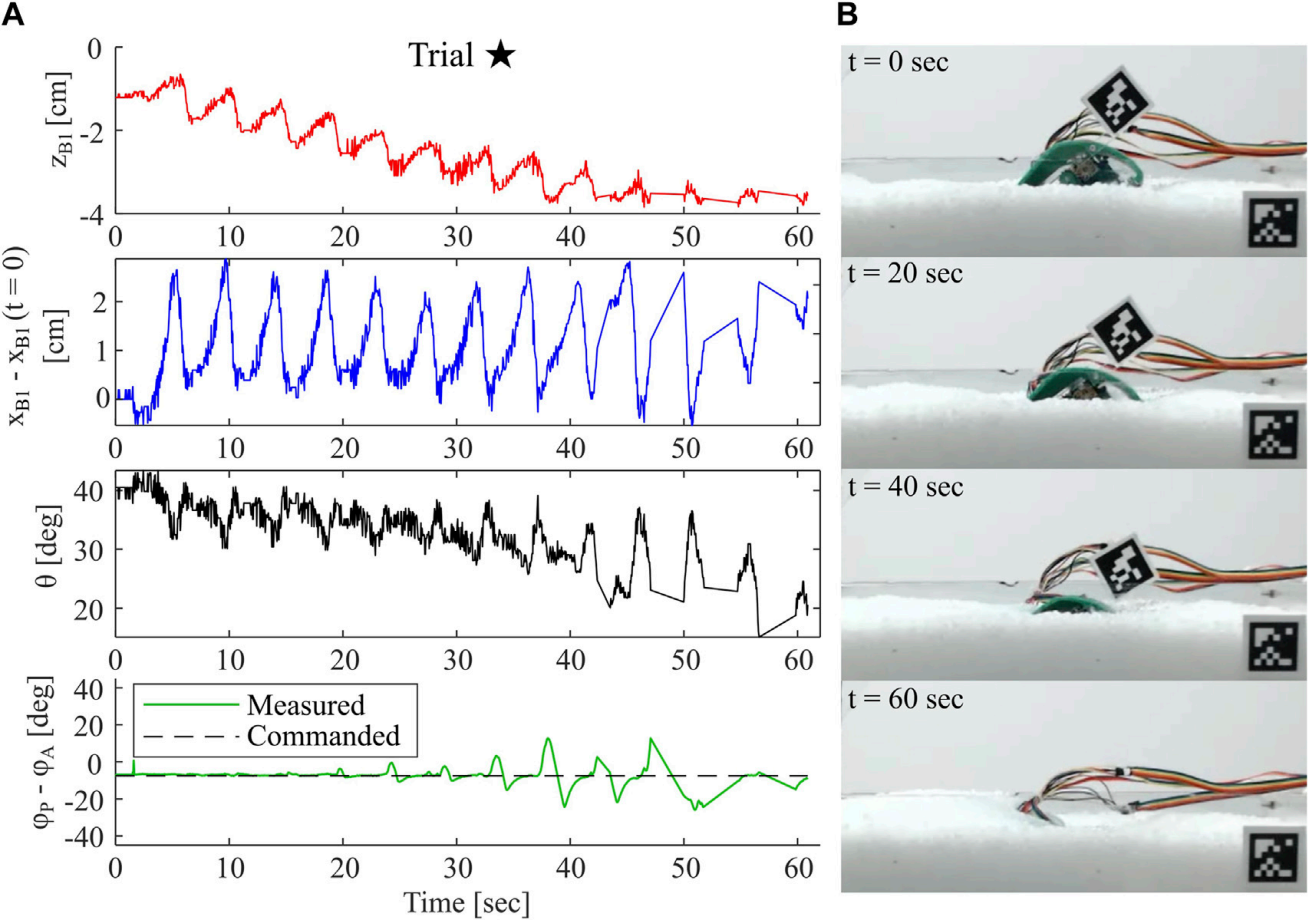
Example of Successful Robot Burrowing Sequence. **(A)** Plots of depth measured relative to substrate surface, horizontal position relative to initial position, sagittal plane body pitch, and measured phase lag between leg pairs throughout a single burrowing sequence **(B)** Images of robot during the corresponding burrowing sequence, with timepoints indicated.

In Figure 12, we show all data collected for Trial ⋆, our deepest recorded burrow. In this case, this successful experimental trial appears to operate in a regime close to the maximum and minimum values presented in Section 3.1, i.e. an initial body pitch of approximately 40-60^*o*^, and phase slightly negative. As shown in the images on the right, the robot and aruco tag completely submerge in the substrate in approximately 60 seconds. The depth data for *z*_*B*1_ indicates an approximately linear but highly oscillatory decrease to near −4 cm. Achieved *z*_*B*1_ likely exceeded this depth, but the true minimum was not captured in the data because the tag became submerged. The horizontal position profile *x*_*B*1_ indicates no strong trends or deviation from the initial position, but does oscillate throughout the burrow with an amplitude of approximately 2 cm. The frequency of oscillations observed in both *z*_*B*1_ and *x*_*B*1_ likely correspond to the frequency of leg cycles, as *z*_*B*1_ decreases sharply during the power stroke and increases slightly during each return stroke. We also plot *θ* throughout the burrow, and note a slight decreasing trend from its initial at approximately 40^*o*^. Compared to most trials in Mode 1, *θ* in this trial remains relatively close to its initial value, possibly permitting the robot to achieve the depths observed here. We also plot both the commanded and measured phase lag, *ϕ*_*P*_ − *ϕ*_*A*_, between leg pairs. At shallow depths, the phase tracks closely to the commanded value, but deviates by as much as 20^*o*^ at greater depths. This deviation is caused by saturation of the motors’ torque capabilities due to the high resistive forces at greater depths. Further tuning of the control algorithm and use of motors with higher torque capabilities would alleviate this error.

## 4 Discussion

EMBUR demonstrates new robotic capability by implementing principles drawn from nature. This legged system is able to burrow itself downward, despite the physically asymmetric forces in granular media that tend to move bodies outward. Our robot provides one solution to this longstanding challenge in the design and control of such robots. At the core of its functionality is a compliant leg design that extends and retracts thought its sweeping cycle and demonstrates highly anisotropic behavior. Two leg pairs whose power strokes move in opposite directions to one another enable downward motion, without pushing the robot out, at intermediate intrusion angles and body pitches of approximately 40–60^*o*^. Because granular RFT modeling predictions suggest upwards net forces, we suspect other factors such as localized material rearrangement and mounding may also be at play in EMBUR’s success. EMBUR uses simplified mechanical and control methods, as compared to the animal, and we suspect that this low degree-of-actuation model results in the sensitivity to initial conditions; Section 3.2.1 demonstrates that < 5 trials achieve full burial out of > 50 trials. The robot’s most successful trials occur when a specific body pitch is initialized and maintained for longer during the burrow, as found in Section 3.2.1. In future work, stability could be analyzed to inform new degrees of freedom and control strategies for maintaining desired body pitch.

### 4.1 Comparisons with biological observations

The design of EMBUR utilized two key observations of the leg morphology and control of *E. analoga* to facilitate burrowing into granular media. First, the inclusion of two leg pairs with oppositely oriented power strokes mimicked the oppositely oriented power strokes of the uropods and leg pair four and leg pairs one to three in the mole crabs, respectively. Second, the retractable leg design (Figure 3C) functioned to reduce drag resistive forces on the legs as they were reset following the power stroke, similarly to the retraction of leg 3 towards the carapace during the limb cycle observed in *E. analoga* (Figure 2C) Using these bioinspired features, EMBUR was able to overcome large substrate resistive forces to fully submerge itself in dry granular media. Successful burrowing trials by EMBUR shared key similarities with the observed burrowing kinematics of *E. analoga*, including an approximately linear vertical descent into the substrate, and approximately constant average horizontal position. These similarities suggest conserved features of the excavation mechanisms used by both EMBUR and *E. analoga*, and consequently more systematic investigation of the mechanics of alternating leg excavation would be an interesting avenue for future work.

We observed several significant differences between the burrowing performance and behavior of EMBUR and the mole crabs that suggest possible directions for future work. First, EMBUR’s burrowing speed was significantly slower than the mole crabs, resulting in an order of magnitude increase in the time required to fully submerge compared to the mole crabs. This discrepancy is likely in part due to the scale difference between the mole crabs and EMBUR, with the significantly larger cross-sectional area of the robot (∼ 31x) resulting in larger substrate resistive forces to be overcome by the burrowing mechanism. The kinematic behavior of EMBUR also deviated from that of the mole crab. We observed the presence of large translational oscillations in both the horizontal and vertical directions during burrowing by EMBUR, compared to the relatively smooth burrowing trajectories of the mole crab. These oscillations likely result from drag forces on the legs during reset, pushing the robot out of the substrate. Combined with the increased burrowing time, these observations suggest that EMBUR’s limb cycles were less effective than those of *E. analoga*. Future modifications to EMBUR could include additional leg pairs to increase excavation capacity, and additional active degrees of freedom in the legs to more closely approximate the spatial retraction trajectories of the mole crab appendages. Minimization of translational oscillations during burrowing, particularly in the vertical direction, may also serve as a useful heuristic for tuning and optimizing leg phase control strategies.

### 4.2 Granular media scaling relations

To draw comparisons between the robot burial and that of the animals, we measure or estimate common metrics for each. EMBUR is measured to be 83x as massive as the mean *E. analoga* specimen and 111x the volume. As a result, the estimated density of each is comparable, ie. EMBUR’s density is 0.74x that of *E. analoga*. However, the insertion force profiles differ more greatly. For this analysis, we utilize the 3D RFT model as in Treers et al. (2021) and measured resistive coefficient *ζ* = 0.111 and 0.401 for the robot and animals’ experimental substrate, respectively. In the 3D RFT model, we insert at 45^*o*^ up to complete burial for the shell of EMBUR as well as an ellipsoid approximation for *E. analoga*. The maximum force magnitude required is 14x greater for the robot than the typical animal size, and 43x the energy expenditure. We then estimate the percent of the required downward force which is accounted for by the mass of the burrowing agent: for EMBUR, weight is 10.4%, while for *E. analoga*, weight is 1.8%. This implies *E. analoga* is highly effective at producing downward forces relative to its own bodyweight, more so than EMBUR. The energy expenditure per unit mass for EMBUR is approximately half that of *E. analoga*.

#### 4.2.1 Limitations and future work

The current robotic system is tested only in grains which are dry and relatively uniform, which is not representative of the animals’ real environment. It is speculated that the saturated media in which *E. analoga* burrows allows it to fluidize the surrounding media, thus lessening the forces required to burrow (Trueman, 1970). The typical beach sands in which *E. analoga* burrows have smaller grain sizes and are typically more dense than the grains we tested EMBUR in, increasing the difficulty of burrowing. In future work, we aim to explore the role of fluidization and saturated media in both robotic and animal behavior to derive more complete functional comparisons with the real animal. We also seek to expand the capability of our models to account for such complex media, while minimizing the discrepancy in both grain size and density between the media used for testing and real sands, and ideally test the robot in field settings.

### 4.3 Digging deeper

Future work will focus on achieving greater depths through a variety of mechanisms. In addition to implementing motors with greater torque capabilities, modifications to leg geometries and material could alleviate stalling at greater depths. New leg designs and trajectories could also be used to target other burrowing strategies such as fluidization, or manipulate the local stress state of the substrate through inertial effects. Additionally, one of the primary benefits of creating a legged burrower is the possibility to use multimodal locomotion. For example, by adding degrees of freedom and additional linkages to the current legs, a combination of walking or running gaits, swimming, and digging modes could be employed. Modification of gaits may also allow for burrow reversal or “burrowing out” behaviors. We also anticipate that achieving such multimodal behaviors may benefit from the incorporation of new leg pairs in addition to the current two used for burrowing. In order to emulate the robustness of the animals’ capability, future work should study leg function in the transition between these different modalities.

## Data Availability

The raw data supporting the conclusions of this article will be made available by the authors, without undue reservation.

## Supplementary material

Supplemental Video S1 Video summarizing major robotic components and experimental results.
